# Dietary Diversity and Healthy Aging: A Prospective Study

**DOI:** 10.3390/nu13061787

**Published:** 2021-05-24

**Authors:** Jian Zhang, Ai Zhao

**Affiliations:** 1 Vanke School of Public Health, Tsinghua University, Beijing 100091, China; zhangjian92@pku.edu.cn; 2 Department of Nutrition and Food Hygiene, School of Public Health, Peking University, Beijing 100191, China

**Keywords:** healthy aging, dietary diversity, physical functional limitation, comorbidity, cognition, psychological stress

## Abstract

Population aging is a global phenomenon. The present study determined the effects of dietary diversity score (DDS) and food consumption on healthy aging. A subset of the data of the China Health and Nutrition Survey was utilized in this study. DDSs were calculated using the dietary data collected in the years 2009 and 2011. A healthy aging score (HAS) was calculated by summing the standardized scores on physical functional limitation, comorbidity, cognitive function, and psychological stress based on the data collected in the year 2015, with a lower HAS indicating a healthier aging process. Life quality was self-reported in the year 2015. This study found that DDS was inversely associated with HAS (T3 vs. T1: β −0.16, 95%CI −0.20 to −0.11, *p*-trend <0.001). The consumption of meat and poultry, aquatic products, and fruits was inversely associated with HAS, and participants in the highest tertile of staple foods consumption had a higher HAS than those in the lowest tertile. HAS was inversely associated with good self-reported life quality and positively associated with bad life quality. In conclusion, food consumption may influence the aging process, and adherence to a diverse diet is associated with a healthier aging process in elderly people.

## 1. Introduction

Population aging is a global phenomenon [1]. In China, people aged 65 years and above made up 13.5% of the total population in 2020 [2] and the proportion is still increasing [3]. Due to the development of the economy and technology, life expectancy continues to increase [4,5]. However, healthy life expectancy increases more slowly than life expectancy [6,7]. It turns out that more older people live in a less healthy state and increasing medical and social resources are needed by the older population [1,6]. Thus, actions to promote a healthier aging process are necessary.

The concept of healthy aging or successful aging was first discussed by Robert J. Havighurst in 1961 [8]. Many of the early definitions of healthy aging were in line with the biomedical model, which was largely based on the absence of disease and disability and classified individuals into healthy and diseased [9,10]. However, as people are living longer, chronic conditions are becoming more common in older people [11], and many individuals have one or more health conditions that are well controlled [12]. In 2009, Young et al. proposed a multidimensional model of healthy aging, emphasizing the coexistence of healthy aging and chronic diseases [13,14]. Young’s model covered three domains: physiological (e.g., diseases and functional impairment), psychological (e.g., emotional vitality and cognitive function), and social (e.g., spirituality and adaptation through social support mechanisms) [13]. This model is flexible, and individuals can succeed in some aspects while having limitations in other aspects [13]. To date, there is still no consensus on what healthy aging should comprise [9,12], but the World Health Organization (WHO) proposed physiological and mental capacities as the intrinsic capacity of individuals and the central part of healthy aging [12].

Nutrition is a key determinant of health and well-being throughout the whole lifecycle [15,16]. Some studies have investigated the association between age-related degenerations and some dietary patterns, such as Mediterranean diet score and diet quality index [17]. However, most of these dietary patterns were developed based on data from Western populations, and some food groups included in these patterns are consumed differently among Chinese populations in terms of quantity and the cooking methods used. Thus, the applicability of these patterns in Chinese populations remains unclear. The dietary diversity score (DDS) is an index used to reflect nutrient adequacy [18]. It is widely used in different populations and across all age groups [18]. A diverse diet is a cornerstone of a sufficient and balanced supply of nutrients. Adherence to a diverse diet is recommended by the WHO [19] and the dietary guidelines for Chinese populations [20]. Our previous work showed that higher diet diversity was associated with better memory status in adults [21]. Moreover, prospective studies have suggested that dietary diversity is inversely associated with all-cause mortality in Chinese and Japanese populations [22,23]. However, evidence of dietary diversity in relation to the overall healthy aging process is scarce in the literature. In the present study, we investigated the association of DDS and food consumption with healthy aging using prospective data from elderly Chinese people.

## 2. Materials and Methods

### 2.1. Study Design and Study Population

**Figure 1:**
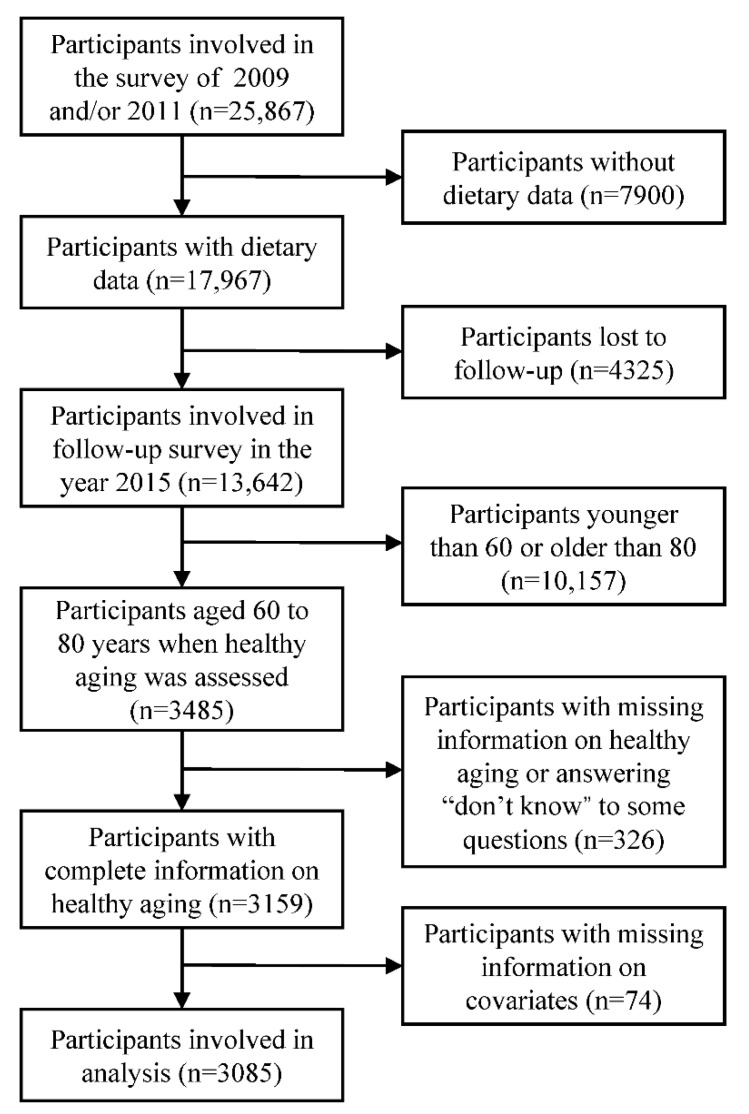
Flow chart of sample selection.

The present study utilized data collected in the China Health and Nutrition Survey (CHNS). The CHNS was a dynamic cohort study. The participants came from twelve geographically diverse areas of China. The first wave of the survey was initiated in 1989, and the last survey—which is open access—was conducted in 2015. Details about the CHNS have previously been published [24]. Prospective data collected in the years 2009, 2011, and 2015 were used in the present study. Baseline information was collected in the years 2009 and 2011, and outcomes (healthy aging) were assessed in the year 2015. The inclusion criteria of the present study included being involved in the year 2009 and/or 2011, involved in the year 2015, and aged 60 to 80 years in the year 2015. The exclusion criteria were having missing information on covariates, being absent from the dietary survey, having missing information on healthy aging indices, answering “don’t know” to any questions concerning physical functional limitation and comorbidity, and answering “don’t know” to all questions concerning psychological stress. Finally, 3085 participants were included in the analysis (Figure 1).

### 2.2. Dietary Survey and Dietary Diversity Score

Dietary data collected in the years 2009 and 2011 were used in this study. A total of 35.2% of participants were involved in one wave of the survey and the others were involved in both surveys. Details about the dietary survey process have been published elsewhere [25]. In brief, dietary intakes during three continuous days were recorded with the methods of dietary recall and household food weight inventory. Energy intake was estimated based on the China food composition table [26,27]. Daily food consumption at each wave of survey was expressed as amount per 1000 kcal. For individuals who participated in both waves of the survey, the average intakes across surveys were calculated.

The DDS was developed according to the dietary guidelines for Chinese populations [20]. The dietary guidelines for Chinese populations give suggestions regarding the consumption of ten food groups, including staple foods (cereals, tubers, and beans), vegetables, fruits, eggs, aquatic products, meat and poultry, soybeans and nuts, milk and dairy products, salt, and oil. As salt and oil are essential parts of the Chinese diet, they were excluded when assessing DDS. In the end, the DDS included eight food groups. The consumption of any food from a certain food group in the past 24 h would add one point for that food group. The DDS ranged from 1 to 8. Our previous study reported that a positive trend between the DDS and intakes of most macronutrients and micronutrients in Chinese adults [21]. The average daily DDS was calculated for each participant at each wave. For participants involved in both dietary surveys in the years 2009 and 2011, the average DDS was calculated. The DDS was then grouped into tertiles from low to high (T1: 1.7–3.3, T2: 3.5–4.3, and T3: 4.4–8.0) for further analysis.

### 2.3. Healthy Aging Score

According to the definition established by Young et al., healthy aging includes three domains: physiological, psychological, and sociological [13]. As the CHNS only covered questions on two of the three domains, the healthy aging score (HAS) in this study was calculated based on physiological and psychological aspects.

The physiological aspect included two indices: physical functional limitation and comorbidity. Physical functional limitation was defined as the number of basic tasks that participants reported difficulty in performing, including lifting a 5 kg bag, squatting down, standing up, sitting continuously, and walking a kilometer. Comorbidity was defined as the number of chronic conditions, including hypertension, diabetes, myocardial infarction, stroke, apoplexy, and asthma. Both previously diagnosed hypertension and systolic blood pressure ≥140 mmHg and/or diastolic blood pressure ≥90 mmHg in the survey were regarded as hypertension. Other medical histories were based on self-reports.

The psychological aspect includes two indices: cognitive function and psychological stress. A subset of tests from the Telephone Interview for Cognitive Status-modified was used to estimate the cognitive function of adults aged 55 years and above in the CHNS [28], including immediate and delayed free-recall test (10 words), the Serial 7s test, and counting backward from 20. Responses to the four questions were summed to give a score ranging from 0 to 27, with a higher score indicating better cognitive function. Psychological stress was estimated by the Perceived Stress Scale (PSS), which has been previously validated in Chinese populations [29]. The PSS uses 14 questions to measure the stress that participants have felt during the last month, e.g., “In the last month, how often have you felt that you were unable to control the important things in your life?”. The answers were recorded on a Likert response scale, consisting of “never”, “almost never”, “sometimes”, “fairly often”, “very often”, and “don’t know”. In the analysis, “don’t know” was replaced by “sometimes”, the neutral midpoint of the Likert scale [30]. The total PSS score was then calculated according to the standard methods [31]. PSS scores ranged from 0 to 56, with a higher score indicating that participants felt more stress.

The HAS was calculated by summing the scores of the four indices mentioned above. Before summation, each index was converted to a continuous distribution between 0 and 1 by dividing the full score of each. The value of cognitive function was reversed before summation. Finally, we obtained a HAS ranging from 0 to 4, with a lower score indicating healthier aging.

### 2.4. Self-Reported Life Quality

Self-reported life quality was surveyed in the year 2015. Participants were asked “How do you rate the quality of your life at present?” (very good, good, fair, bad, very bad, and don’t know). Participants who chose “don’t know” were excluded. We grouped the participants’ life quality into good (very good/good), fair, and bad (bad/very bad) for further analysis.

### 2.5. Covariates

Information on covariates of interest was obtained from the baseline survey, including sociodemographic characteristics (age, gender, region of residence, residency, education, household income, and marriage status), lifestyle behaviors (smoking and alcohol use), and anthropometric measurements (weight and height).

Per capita household income was grouped into tertiles and labeled as low, middle, and high at each wave of the survey. Missing values for income were imputed by the medians of each site at each wave of the survey. Body mass index (BMI) was calculated as weight/(height)^2^.

### 2.6. Statistics

Results were presented as means and standard deviations for normally distributed variables; otherwise, medians and interquartile ranges (IQR) were presented. Categorical variables were presented as percentages. ANOVA and Chi-square tests were performed to compare the differences across DDS tertiles for normally distributed continuous variables and categorical variables, respectively.

The association between DDS tertiles and HAS was investigated with linear regression models; association between DDS tertiles and healthy aging components (physical functional limitation, comorbidity, cognitive function, and psychological stress) were investigated by ordinal logistic regression models. Physical functional limitation and comorbidity were grouped into categorical variables according to participants’ number of limitations or comorbidities (0, 1–2, or ≥3). Cognitive function and psychological stress scores were grouped into tertiles from low to high. Multivariate models were adjusted for age (years), gender (men or women), BMI (kg/m^2^), region of residence (southern or northern China), residency (rural or urban), education (primary school and below or middle school and above), income (low, middle, or high), marriage status (married or others (divorced, widowed, separated, or never married)), smoking (current smoker or not), and alcohol use (≥1 or <1 time per week).

We further investigated food group consumption in relation to HAS. Data on food consumption were converted into categorical variables, then linear regression models were created. For food groups with a relatively low proportion of consumers (milk and dairy products, aquatic products, and fruits), the daily average food consumption was grouped into non-consumer, low (below the median in consumers), and high (above the median in consumers). Other food groups were grouped into tertiles and labeled as low, middle, and high. The covariates mentioned above were also adjusted in the analysis.

Multinomial logistic regression models were created to investigate the association of HAS with self-reported life quality (fair, good, or poor).

Subgroup analyses of the association between DDS tertiles and healthy aging were conducted according to age (≤60 or >60 years), gender (man or women), region of residence (southern or northern China), BMI (<24 or ≥24 kg/m^2^), smoking (current smoker or not), and alcohol use (≥1 or <1 time per week). Interaction was tested by likelihood ratio tests, which compared the model with and without interaction terms between DDS tertiles and the baseline stratifying variables. Sensitivity analysis was conducted by excluding participants who answered “don’t know” to at least one of the questions in the assessment of psychological stress.

Tests for linear trends across categories were conducted by assigning the medians of each category and treating the variable as continuous in a separate regression model. All the statistics were analyzed using R 4.0.5 (R Core Team, Vienna, Austria). Additional packages were used for ordinal logistic regression (MASS [32]), multinomial logistic regression (nnet [32]), and likelihood ratio test (lmtest [33]). All *p* values were two-sided, and statistical significance was defined as *p* < 0.05.

## 3. Results

### 3.1. Baseline Characteristics

The baseline characteristics of participants across DDS tertiles are shown in Table 1. Participants with a higher DDS had a higher BMI, had higher proportions of people living in southern China and urban areas, had higher levels of education and per capita household income, were more likely to be married, and had a lower proportion of smokers.

### 3.2. Dietary Diversity Score and Healthy Aging

The average HAS of the participants was 1.26 ± 0.50. The DDS was inversely associated with HAS after the adjustment of covariates (a lower HAS indicated healthier aging). Higher DDS was associated with better cognitive function, fewer physical functional limitations, and less psychological stress. The association between DDS and the number of comorbidities was insignificant (Table 2).

**Figure 2:**
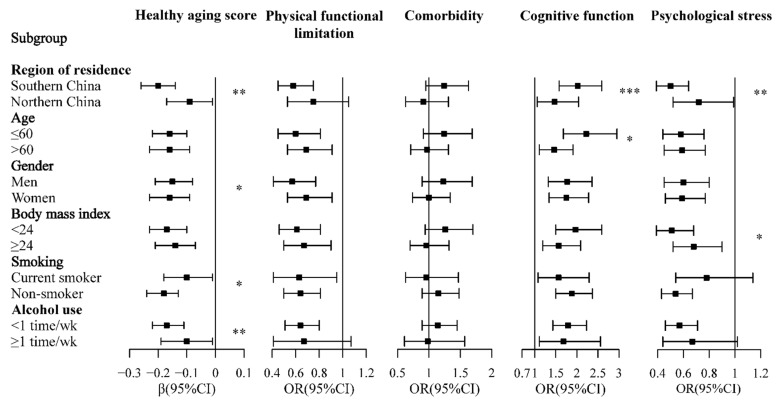
Subgroup analysis of the association between dietary diversity score tertiles and healthy aging. Dietary diversity scores were grouped into tertiles from low to high (T1: 1.7–3.3, T2: 3.5–4.3, and T3: 4.4–8.0). Squares and horizontal lines showed the results of T3 vs. T1. * *p* < 0.05, ** *p* < 0.01, *** *p* < 0.001. OR, odds ratio; CI, confidence interval. Association of DDS tertiles with healthy aging score (lower score indicated healthier aging) was estimated by a linear regression model. Association of DDS tertiles with physical functional limitation, comorbidity, cognitive function, and psychological stress was estimated by an ordinal logistic regression model. Comorbidity and physical functional limitations were classified into categorical variables according to the number of comorbidities or functional limitations (0, 1–2, or ≥3). Cognition and PSS were grouped into tertiles from low to high. Multivariate models were adjusted for age, gender, region of residence, residency, education, income, marriage status, body mass index, smoking, and alcohol use. Analyses within subgroups were adjusted for all other covariates.

Significant interactions were observed between DDS tertiles and gender, region of residence, smoking status, and alcohol use in relation to HAS (Figure 2). The association of DDS tertiles with cognitive function was modified by age and region of residence, while the association of DDS tertiles with psychological stress was modified by BMI and region of residence (Figure 2).

In the sensitivity analysis, the association of DDS with HAS and psychological stress did not change appreciably after excluding participants who answered “don’t know” in the assessment of psychological stress (Table S1).

### 3.3. Food Group Consumption and Healthy Aging

The consumption of meat and poultry, aquatic products, and fruits was inversely associated with HAS. Compared with non-consumers, a low consumption of milk and dairy products was associated with a lower HAS. Participants in the highest tertile of staple foods consumption had a higher HAS than those in the lowest tertile. The association of the consumption of eggs and HAS became insignificant after the adjustment of covariates. There was no significant association between HAS and the consumption of vegetables and the consumption of soybeans and nuts (Table 3).

### 3.4. Healthy Aging Score and Self-Reported Life Quality

The association of HAS with self-reported life quality is shown in Table 4. HAS was inversely associated with good self-reported life quality and positively associated with bad life quality.

## 4. Discussion

Our study revealed that the consumption of some food groups may impact the aging process and that a more diverse diet contributed to a healthier aging process in older adults. Moreover, healthy aging was associated with good self-reported life quality. To the best of our knowledge, the present study is the first to explore the association of DDS with overall healthy aging. The results confirm that the adoption of a diverse diet may provide a cost-effective intervention to promote healthy aging.

Our study found that higher DDS was associated with overall healthy aging in elderly Chinese people. The analyses of DDS in relation to healthy aging components showed that a higher DDS was associated with better cognitive function and fewer physical functional limitations and psychological stress, which was consistent with results from the literature. Prospective studies have proven that an increase in DDS is associated with reduced risk of cognitive impairment in elderly Chinese and Japanese people [34,35], and previous studies have also indicted that having a higher dietary diversity is associated with better physical function in elderly Japanese people [36]. We assumed that several pathways might contribute to the association between DDS and healthy aging. First, the aging process is characterized by a loss of muscle mass and strength [37], increasing the risks of functional impairment [38], disability [39], and frailty [40]. Sufficient protein intake is good for preserving good muscle function in older people [41,42]. Our previous work showed that higher DDS was associated with a higher intake of protein in Chinese adults [21], which may slow down the loss of muscle mass caused by aging. The analysis of food group consumption and healthy aging supported this assumption, which showed that higher intakes of meat and aquatic products, both good sources of high-quality protein, were inversely associated with HAS. Second, older people usually experience a degeneration in their physical and mental capability, including a decrease in mobility, degeneration of the digestive system, and decline in income, causing them to be at higher risks of malnutrition [43,44,45]. Higher DDS was reported to be inversely associated with the risk of nutrient inadequacy [46,47], which may prevent health problems related to insufficient nutrient intake. Third, inflammation and oxidative stress are thought to be one of the major causes of aging, accelerating the loss of muscle and bone mass and the degeneration of the function of the central nervous system [48,49,50,51]. As vegetables and fruits are good sources of antioxidants, a higher DDS may promote healthier aging by reducing inflammation and oxidative stress [52]. Last but not least, it is suggested that age-related gut microbiota dysbiosis can trigger the innate immune response and chronic low-grade inflammation, leading to frailty and unhealthy aging [53,54]. A diverse diet was reported to promote a healthier gut microbiota [55], which may further promote healthy aging [56].

Specifically, psychological stress is an important aspect of wellbeing, and stress has been reported to play an important role in the development of depression [57], which is an increasing social concern in elderly Chinese people [58]. Previous studies have reported that psychological stress may be a risk factor for chronic diseases (e.g., diabetes and cardiovascular diseases) [59,60]. As China is experiencing a fast transition in technology, economy, and lifestyle, older people may face more stress from these changes [61,62]. The impact of diet on psychological stress is relatively difficult to investigate, as the association is bidirectional [63]. However, it has been proven by clinical trials that the Mediterranean diet is protective against depression and psychological problems [64,65]. Our prospective study found that higher DDS was associated with lower PSS scores in elderly Chinese people. The beneficial effect of higher DDS on psychological stress may be related to the elevated intake of antioxidants from a diverse diet [66]. Moreover, recent findings on the gut–brain axis have suggested that maintaining a healthy microbiota might be important for mental health [63,67], thus a higher DDS might also contribute to better mental health through a healthier gut microbiota.

Regarding food groups, the present study found that participants in the highest tertile of staple foods consumption had higher HAS than those in the lowest tertile while there was no significant difference between participants in the lowest and middle tertiles. Staple foods were the major source of carbohydrates in the Chinese diet. Evidence in the literature has suggested that high carbohydrate intake might have negative effects on mental health. Prospective studies have reported that dietary patterns characterized by high intake of protein and fat and low intake of carbohydrate were associated with lower risks of cognitive impairment and dementia [68,69]. Some other studies have reported that a low-carbohydrate diet was associated with lower risks of depression in Iranian women [70] and patients with diabetes [71]. Moreover, we propose that another possible reason for this association may be that individuals with a high consumption of staples foods have a lower score for DDS in the study population.

In the subgroup analyses, we observed that the association of DDS with HAS was less pronounced in individuals who were current smokers or who drank alcohol ≥1 time per week compared to their counterparts. Smoking generates radicals and increases inflammation, causing lipid peroxidation and the oxidation of proteins [51,72,73]. It has been recognized as one of the most important risk factors for respiratory diseases, cardiovascular diseases, and cancers, which are the major causes of disability and mortality [74,75]. Alcohol use is another leading risk factor for disease burden and health loss [76], increasing the risk of hypertension [77], liver cancer [78], and gastrointestinal cancer [79]. Thus, smoking and alcohol use might attenuate the positive association between DDS and healthy aging. Moreover, our study found that the association between DDS and healthy aging was more pronounced among individuals living in southern China than those living in northern areas. This may be because the northern and southern populations of China have some differences in terms of lifestyle, diet, and the environment. According to previous studies, the northern dietary pattern is more likely to contribute to the development of overweight and obesity [80], and the northern population is more likely to have clustered cardiovascular risk factors than the southern population [81].

In addition, the present study found that higher HAS was negatively associated with self-reported good life quality and positively associated with bad life quality. This is reasonable, as a higher HAS means more physical functional limitation, comorbidity, psychological stress, and poor cognitive function. The results suggest that improving dietary diversity may be regarded as one of the strategies to promote healthy aging and to improve the life quality of elderly people.

When constructing a DDS, the food groups included were different across studies, partly due to the study population and purpose [18,82,83]. In the literature, the five-food-group DDS according to the USDA Food Guide Pyramid or a modified version of the score have been used relatively more frequently [82,83]. In the present study, we calculated the DDS based on the dietary guidelines for Chinese populations [20], which additionally includes some food groups which play important roles in the Chinese diet.

The present study had several potential limitations. First, although the present study was based on a prospective setting, participants’ baseline information regarding outcomes was not taken into consideration, as most of these indices were not included in the baseline survey. This may impact the causal inference in this study. Second, the definition of healthy aging and the indices included in the estimation of healthy aging were different across studies. We calculated the healthy aging score based on the data available, which may impact comparison with other studies. In the present study, the HAS was constructed based on participants’ physiological and psychological conditions, and the sociological aspect was not included. Thus, the association of dietary diversity and healthy aging was not comprehensively investigated. However, as physiological and mental capacities were regarded as the intrinsic capacity of individuals according to the WHO report [12], this work could contribute to our knowledge on dietary intake and healthy aging. Further studies in regard to sociological aspects of aging are still needed to provide a more comprehensive picture.

## 5. Conclusions

In summary, we found that the consumption of some food groups may impact the aging process and adherence to a more diverse diet was associated with a healthier aging process in elderly people. Higher DDS was associated with better cognitive function, fewer physical functional limitations, and less psychological stress. This study confirmed that the adoption of a diverse diet may provide a cost-effective intervention for the promotion of healthy aging.

## Figures and Tables

**Table 1:** Baseline characteristics of the participants across dietary diversity score tertiles.

Variables	Dietary Diversity Score ^a^			*p*
	T1	T2	T3	
Number of participants	991	1076	1018	
Age (year)	62.1(5.6)	61.6(5.5)	61.9(5.6)	0.088
Body mass index (kg/m^2^)	23.3(3.8)	23.9(3.4)	24.4(3.2)	<0.001
Gender				0.166
Men	44.1	48.2	46.7	
Women	55.9	51.8	53.3	
Region of residence				<0.001
Southern China	60.9	69.1	63.0	
Northern China	39.1	30.9	37.0	
Residency				<0.001
Rural	84.2	65.1	36.1	
Urban	15.8	34.9	63.9	
Education				<0.001
Primary school and below	80.7	61.9	35.1	
Middle school and above	19.3	38.1	64.9	
Per capita household income				<0.001
Low	54.0	32.4	11.8	
Middle	31.1	37.7	25.3	
High	14.9	29.9	62.9	
Marriage status				<0.001
Married	83.9	88.4	90.2	
Others	16.1	11.6	9.8	
Smoking				0.001
Current smoker	30.1	28.0	23.1	
Non-smoker	69.9	72.0	76.9	
Alcohol use (times/week)				0.474
<1	79.8	78.0	77.8	
≥1	20.2	22.0	22.2	

^a^ Dietary diversity scores were grouped into tertiles from low to high (T1: 1.7–3.3, T2: 3.5–4.3, and T3: 4.4–8.0). Continuous variables are presented as means and standard deviations; categorical variables are presented as percentages. Continuous variables were compared across dietary diversity score tertiles by ANOVA; categorical variables were compared by Chi-square tests.

**Table 2:** Association of dietary diversity score with healthy aging.

Variables	Dietary Diversity Score ^a^			*p*-Trend
	T1	T2	T3	
Healthy aging score ^b^				
Crude	Ref	−0.12(−0.16, −0.08)	−0.26(−0.31, −0.22)	<0.001
Model 1	Ref	−0.06(−0.10, −0.02)	−0.15(−0.20, −0.11)	<0.001
Model 2	Ref	−0.06(−0.10, −0.02)	−0.16(−0.20, −0.11)	<0.001
Physical functional limitation ^c^				
Crude	Ref	0.77(0.65, 0.90)	0.58(0.49, 0.68)	<0.001
Model 1	Ref	0.87(0.73, 1.04)	0.64(0.52, 0.79)	<0.001
Model 2	Ref	0.86(0.73, 1.03)	0.64(0.52, 0.78)	<0.001
Comorbidity ^c^				
Crude	Ref	1.07(0.90, 1.27)	1.26(1.06, 1.50)	0.010
Model 1	Ref	1.09(0.91, 1.31)	1.19(0.97, 1.47)	0.101
Model 2	Ref	1.01(0.84, 1.22)	1.09(0.88, 1.35)	0.441
Cognitive function ^c^				
Crude	Ref	1.58(1.34, 1.85)	2.88(2.44, 3.39)	<0.001
Model 1	Ref	1.25(1.05, 1.48)	1.80(1.48, 2.19)	<0.001
Model 2	Ref	1.23(1.04, 1.46)	1.77(1.46, 2.15)	<0.001
Psychological stress ^c^				
Crude	Ref	0.56(0.48, 0.66)	0.42(0.36, 0.50)	<0.001
Model 1	Ref	0.63(0.53, 0.74)	0.59(0.48, 0.71)	<0.001
Model 2	Ref	0.63(0.53, 0.75)	0.59(0.49, 0.72)	<0.001

Ref, reference. ^a^ Dietary diversity scores were grouped into tertiles from low to high (T1: 1.7–3.3, T2: 3.5–4.3, and T3: 4.4–8.0). ^b^ Linear regression models were created to estimate the association of dietary diversity score with healthy aging score; values are β (95% confidence intervals) unless specified. ^c^ Ordinal logistic regression models were created to estimate the association of dietary diversity score with physical functional limitation, comorbidity, cognitive function, and psychological stress; values are odds ratios (95% confidence intervals) unless specified. Comorbidity and physical functional limitations were classified into categorical variables according to the number of comorbidities or functional limitations (0, 1–2, or ≥3). Scores for cognitive function and psychological stress are grouped into tertiles from low to high. Multivariate models were adjusted for: model 1: age, gender, region of residence, residency, education, income, and marriage status; model 2: additionally included body mass index, smoking, and alcohol use.

**Table 3:** Association between food consumption and healthy aging score.

Food Groups	Number of Participants	Median (IQR)	Crude		Adjusted	
			β (95%CI)	*p*-Trend	β (95%CI)	*p*-Trend
Staple foods				<0.001		0.060
Low	1028	130.8 (33.2)	Ref		Ref	
Middle	1028	178.2 (20.5)	0.05 (0.01, 0.10)		0 (−0.04, 0.04)	
High	1029	228.2 (41.5)	0.12 (0.08, 0.16)		0.04 (0, 0.08)	
Soybeans and nuts				0.180		0.995
Low	1028	0 (2.2)	Ref		Ref	
Middle	1028	8.4 (4.1)	−0.05 (−0.09, −0.01)		−0.03 (−0.07, 0.01)	
High	1029	21.2 (13.5)	−0.03 (−0.08, 0.01)		0 (−0.04, 0.04)	
Vegetables				0.585		0.167
Low	1028	95.8 (33.5)	Ref		Ref	
Middle	1028	149.6 (26.2)	0 (−0.05, 0.04)		−0.01 (−0.05, 0.02)	
High	1029	225.0 (70.2)	−0.01 (−0.06, 0.03)		−0.03 (−0.07, 0.01)	
Fruits				<0.001		<0.001
Non-consumer	1482	0	Ref		Ref	
Low	802	31.2 (20.2)	−0.10 (−0.14, −0.06)		−0.05 (−0.09, −0.01)	
High	801	88.1 (54.0)	−0.13 (−0.18, −0.09)		−0.09 (−0.13, −0.05)	
Meat and poultry				<0.001		0.047
Low	1028	16.1 (24.0)	Ref		Ref	
Middle	1028	42.7 (12.2)	−0.08 (−0.13, −0.04)		−0.04 (−0.08, 0)	
High	1029	76.9 (30.6)	−0.13 (−0.17, −0.08)		−0.04 (−0.09, 0)	
Aquatic products				<0.001		<0.001
Non-consumer	1436	0	Ref		Ref	
Low	824	13.8 (8.9)	−0.12 (−0.16, −0.07)		−0.05 (−0.09, −0.01)	
High	825	38.0 (21.7)	−0.17 (−0.21, −0.13)		−0.10 (−0.14, −0.06)	
Eggs				0.006		0.113
Low	1028	0(5.1)	Ref		Ref	
Middle	1028	14.6 (5.7)	−0.02 (−0.06, 0.02)		0.01 (−0.03, 0.05)	
High	1029	30.7 (15.3)	−0.06 (−0.10, −0.02)		−0.03 (−0.07, 0.01)	
Milk and dairy products				<0.001		0.054
Non-consumer	2493	0	Ref		Ref	
Low	296	45.9 (30.3)	−0.13 (−0.19, −0.06)		−0.08 (−0.14, −0.02)	
High	296	126.2 (65.4)	−0.13 (−0.19, −0.07)		−0.05 (−0.11, 0.01)	

IQR, interquartile range; CI, confidence interval; Ref, reference. Consumption of fruits, aquatic products, and milk and dairy products were grouped into non-consumer, low (below the median in consumers), and high (above the median in consumers). Other food groups were grouped into tertiles and labeled as low, middle, and high. Linear regression models were created to estimate the association of food consumption with healthy aging score (a lower score indicates healthier aging). Multivariate models were adjusted for age, gender, region of residence, residency, education, income, marriage status, body mass index, smoking, and alcohol use.

**Table 4:** Healthy aging score and self-reported life quality ^a^.

Self-Reported Life Quality	Number of Participants	Crude		Adjusted	
		OR (95%CI)	*p*	OR (95%CI)	*p*
Fair	1148	Ref		Ref	
Good	1763	0.38 (0.32, 0.44)	<0.001	0.38 (0.32, 0.45)	<0.001
Poor	168	4.15 (3.05, 5.66)	<0.001	4.41 (3.12, 6.25)	<0.001

OR, odds ratio; CI, confidence interval; Ref, reference. ^a^ 3079 participants were included in analysis. Multinomial logistic regression models were created to estimate the association of healthy aging score (a lower score indicates healthier aging) with self-reported life quality. Multivariate models were adjusted for age, gender, region of residence, residency, education, income, and marriage status.

## Data Availability

The raw data supporting the conclusions of this article can be found here: https://www.cpc.unc.edu/projects/china/ (accessed on 23 May 2019).

## Supplementary Materials

The following are available online at https://www.mdpi.com/article/10.3390/nu13061787/s1, Table S1: Sensitivity analysis of the association between dietary diversity score and healthy aging.
