# Supplementary material for: Dietary Diversity and Healthy Aging: A Prospective Study
Source: Nutrients. 2021 May 24;13(6):1787. doi: 10.3390/nu13061787 (PMC8225052; doi:10.3390/nu13061787)
Supplement: Supplementary file 1 [file nutrients-13-01787-s001.zip › nutrients-1230977-supplementary.pdf]

Table S1. Sensitivity analysis of the association between dietary diversity score and healthy aging [a]

Variables	Dietary diversity score [b]			*p*-Trend
	**T1**	**T2**	**T3**	
Number of participants	861	926	891	
Dietary diversity score	3.0(1.7–3.3)	4.0(3.5–4.3)	5.0(4.5–8.0)	
Healthy aging score [c]				
Crude	Ref	−0.14(−0.18, −0.09)	-0.28(−0.33, -0.23)	<0.001
Model 1	Ref	−0.07(−0.12, −0.03)	-0.17(−0.22, -0.12)	<0.001
Model 2	Ref	−0.08(−0.12, −0.03)	-0.18(−0.23, -0.13)	<0.001
Psychological stress [d]				
Crude	Ref	0.52(0.44,0.61)	0.42(0.35,0.50)	<0.001
Model 1	Ref	0.58(0.48,0.69)	0.55(0.45,0.68)	<0.001
Model 2	Ref	0.58(0.48,0.69)	0.56(0.45,0.68)	<0.001

Ref, reference. [a] Participants who answered "don't know" to any questions in the assessment of psychological stress were excluded. [b] Dietary diversity scores were grouped into tertiles from low to high. [c] Linear regression models were conducted to estimate the association of dietary diversity score with healthy aging score; values are β (95% confidence intervals) unless specified. [d] Ordinal logistic regression models were conducted to estimate the association of dietary diversity score with psychological stress; values are odds ratios (95% confidence intervals) unless specified. The scores on psychological stress were grouped into tertiles from low to high. Multivariate models were adjusted for: model 1: age (years), gender (men or women), region of residence (southern or northern China), residency (rural or urban), education (primary school and below or middle school and above), income (low, middle, or high), marriage status (married or others (divorced, widowed, separated, or never married)); model 2: additionally included body mass index (kg/m^2), smoking (current smoker or not), and alcohol use (≥1 or <1 time per week).
